# Sensitive Characterization of the Graphene Transferred onto Varied Si Wafer Surfaces via Terahertz Emission Spectroscopy and Microscopy (TES/LTEM)

**DOI:** 10.3390/ma17071497

**Published:** 2024-03-26

**Authors:** Dongxun Yang, Jesse Henri Laarman, Masayoshi Tonouchi

**Affiliations:** 1 Institute of Laser Engineering, Osaka University, Osaka 565-0871, Japan; 2 Department of Applied Physics, Eindhoven University of Technology, 5612 AZ Eindhoven, The Netherlands; jesselaarman@gmail.com

**Keywords:** graphene, terahertz emission spectroscopy (TES), BHF etching, silicon

## Abstract

Graphene shows great potential in developing the next generation of electronic devices. However, the real implementation of graphene-based electronic devices needs to be compatible with existing silicon-based nanofabrication processes. Characterizing the properties of the graphene/silicon interface rapidly and non-invasively is crucial for this endeavor. In this study, we employ terahertz emission spectroscopy and microscopy (TES/LTEM) to evaluate large-scale chemical vapor deposition (CVD) monolayer graphene transferred onto silicon wafers, aiming to assess the dynamic electronic properties of graphene and perform large-scale graphene mapping. By comparing THz emission properties from monolayer graphene on different types of silicon substrates, including those treated with buffered oxide etches, we discern the influence of native oxide layers and surface dipoles on graphene. Finally, the mechanism of THz emission from the graphene/silicon heterojunction is discussed, and the large-scale mapping of monolayer graphene on silicon is achieved successfully. These results demonstrate the efficacy of TES/LTEM for graphene characterization in the modern graphene-based semiconductor industry.

## 1. Introduction

Graphene, a remarkable two-dimensional carbon material, consists of a single-atom-thick planar sheet of carbon atoms arranged in a honeycomb lattice crystal structure [1]. Each carbon atom forms covalent bonds within the plane, establishing σ-bonds with three neighboring carbon atoms and one out-of-plane π-bond. The unique electronic structure of graphene resembles that of a benzene ring, featuring three occupied bonds and three unoccupied anti-bonds. This arrangement results in some overlap between the valence and conduction bands, giving rise to a band structure characterized by a Dirac cone, which endows graphene with exceptional properties, such as high electron mobility, superb electrical conductivity, and excellent thermal conductivity. These properties make graphene a promising material for electronic devices [2,3,4,5,6,7].

However, the practical application of graphene in real devices often encounters limitations stemming from the variability in its electronic and mechanical characteristics, primarily influenced by its interaction with the underlying substrate. The high flexibility of graphene in the out-of-plane direction means that its morphology is significantly influenced by the geometry of the substrate upon which it is placed. These substrate-induced changes impact the measured properties of graphene, affecting its electronic structure, presence of topological defects, and susceptibility to chemical doping effects [8,9,10,11]. Therefore, the substrate engineering of graphene to achieve high-performance graphene-based devices has emerged as a hot research direction in graphene engineering and application [12,13]. The influence of the substrate surface conditions on graphene properties is necessary to consider and estimate in the application.

The real implementation of graphene-based electronic devices needs to be compatible with existing silicon-based nanofabrication processes. The connection between graphene and Si wafers is inevitable during the fabrication process, and, thus, it is essential to estimate the influence of different Si wafer surfaces. Given that graphene is an atom-thick monolayer of carbon [14], the common techniques for the detection of its presence are imperative. While typical techniques, such as scanning electron microscopy (SEM), transmission electron microscopy (TEM), atom force microscopy (AFM), scanning tunneling microscope (STM), X-ray diffraction (XRD), and Raman spectrum, have been used to characterize the quality, number of layers, defects, and atomic structures of graphene in a small-scale range (from several micrometers to nanometers) [15,16,17,18,19,20,21], they are not suitable for evaluating the substrate influence on graphene properties directly in a large-scale range. New techniques for rapid and non-invasive evaluation of the graphene/Si interface are urgently required. Laser terahertz (THz) emission techniques including spectroscopy and microscopy (TES/LTEM) have shown great potential in achieving the goal of such a mission and characterizing the semiconductor materials [22,23], devices [24,25], and 2D films [26], such as graphene on the Si surface. Ultrafast charge transport leads to THz radiation [27,28,29,30,31]. When the ultrafast laser pulse generates the photocarriers at the Si surface due to its higher photon energy compared to the band gap of the semiconductor, the initial electric field or the gradient of carrier density will accelerate the carriers to move at an ultrafast speed, and the recombination of these photocarriers occurs after several hundred femtoseconds, which leads to microwave radiation within the THz range. The amplitude of the THz emission field is defined by,

(1) $$\;E_{THz}\propto \frac{dJ}{dt},$$

where $E_{THz}$ is the THz emission field, and *J* is the density of the induced photocurrent.

According to the types of ultrafast charge transport, the source of the THz emission from a semiconductor can be concluded as (1) surge drift current [32,33,34]; (2) diffusion current or photo-Dember effect [35,36,37]; (3) ballistic [38]. Based on the basic THz emission mechanism from the semiconductor surface or interface, the electric properties variation, such as electric field, surface/interface potential, surface charges, and defect concentration, can be observed directly or indirectly from the THz radiation waveform, and the parameters of the semiconductor devices can be estimated and extracted quantitatively. In previous research, we successfully estimated the Si surface potential with the influence of surface dipole via THz emission techniques [30]. Furthermore, quantitative estimation of the work function of VO_2_ films in different phase conditions has been achieved by TES [39]. The estimation of monolayer graphene via THz emission techniques is accessible and promising but has not been reported yet.

**Figure 1:**
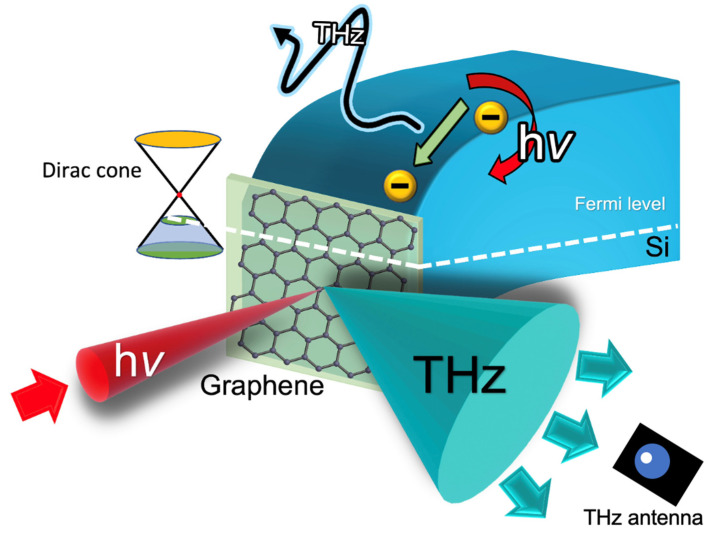
Illustration of THz emission mechanism from semiconductor surface with laser excitation. The THz emission is generated from the ultrafast photoelectrons transport within the band bending (shown as the black THz arrays) and the THz radiation from the laser-illuminated area is shown as the green cone and detected by the THz antenna.

In this study, we explore the use of laser terahertz emission microscopy (LTEM) as a new technique for characterizing the large-scale chemical vapor deposition (CVD) monolayer graphene on silicon substrates [40], as shown in Figure 1. We prepared different doping Si substrates and the buffered hydrogen fluoride (BHF) to remove the native oxide layer on the Si surface before graphene transfer [41]. The roughness of the Si surface after BHF etching was about 1 nm over a distance of several micros, which hardly influences the transferred graphene properties and can be neglected [42]. With the detection of THz emission from graphene/Si samples, the influence of the doping conditions and Si surface conditions has been observed, and large-scale mapping of the graphene on the Si has been achieved.

## 2. Materials and Methods

### 2.1. Sample Preparation

**Figure 2:**
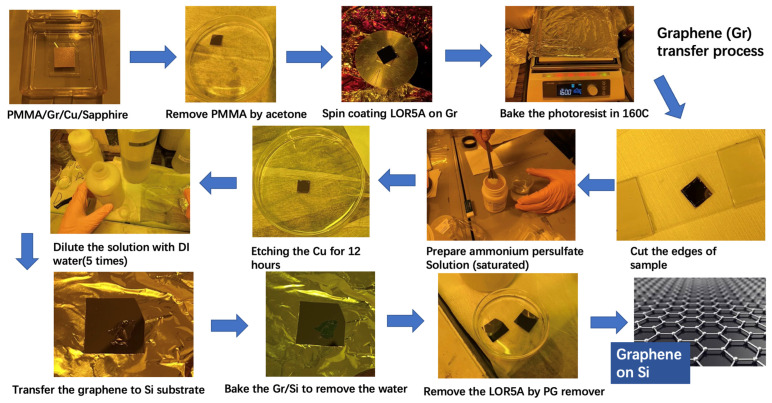
The procedure of graphene transferring onto the Si substrates.

In our experiment, CVD graphene was transferred onto silicon substrates for TES measurement. We prepared both n-type and p-type Si substrates, whose properties are listed in Table 1. The size of each Si substrate was 20 × 20 mm. In addition, we prepared some other Si substrates treated with BHF for 1 min to remove the native oxide layer. The commercial CVD graphene samples were used and transferred to the Si substrate. The original graphene was grown on a copper foil attached to an Al_2_O_3_ substrate. The sample surface was coated with the PMMA protection layer. Figure 2 shows the procedure of the process for transferring CVD graphene films from the original substrate onto a Si substrate by using a PMGI-based photoresist (LOR5A from MicroChem, Tokyo, Japan).

The operation steps are as follows [43]: (1) Removal of the initial PMMA protection layer. First, the original graphene sample is submerged in the acetone solution for 30 min and 10 min in two subsequent dishes to remove the PMMA protection layer properly. Then, the sample is cleaned with deionized (DI) water. Next, the surface of the sample is blown using N_2_ gas flow to remove the water. (2) Spin coating of PMGI resist on graphene surface. The PMGI resist is coating on the surface of graphene for transferring by using a spin coater (MIKASA, Tokyo, Japan). First, the photoresist is dropped on the surface and fully covers the graphene. Then, the sample is rotated at 2000 rpm for 1 min. After spin coating, the sample will be soft baked at 160 °C for 5 min to evaporate cyclopentanone, which is a solvent in LOR photoresist. (3) Etching of the copper foil. To transfer the graphene to the target substrate, we need to etch the copper foil between the graphene and the Al_2_O_3_ substrate and make them detached. First, the edges of graphene are cut by the blade to avoid unintentional attachment. Then, the sample is submerged in the ammonium persulfate-saturated solution for etching the copper foils. The ammonium persulfate powder is purchased from Sigma-Aldrich, Tokyo, Japan. After 6 h of etching, all the copper foil will be removed, and the PMGI/graphene membrane will become free-floating. (4) Transferring graphene onto the Si substrate. To avoid the influence of the acid on the properties of graphene, the acid solution needs to be diluted by DI wafter before transferring the graphene. After multiple dilutions, the Si substrate will be placed under the floating PMGI/graphene membrane and carefully lifted out of the solvent with the graphene on top. Next, the Si substrate with the PMGI/graphene membrane will be baked at 100 °C for 5 min to evaporate the solution between the graphene and Si surface and make the graphene evenly on the surface. (5) Removal of the PMGI layer. The PMGI layer is removed by using the PG remover at 60 °C for 10 min. After that, the PG remover is washed away by using ethanol and DI water subsequently. Finally, the sample surface will be dried by using N_2_ gas flow.

### 2.2. Experimental Setup

**Figure 3:**
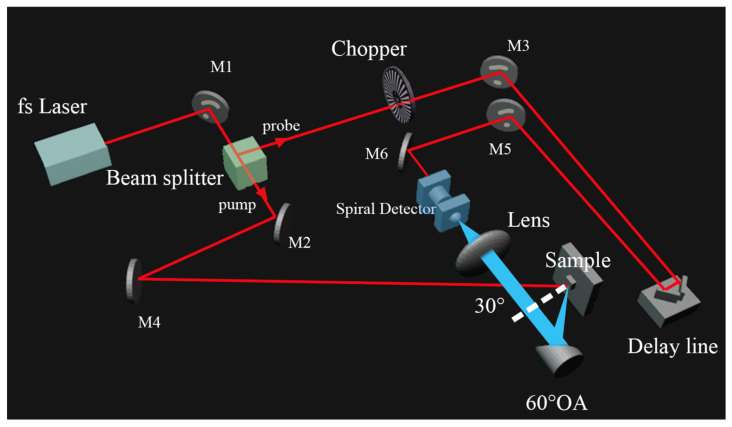
Diagram of the TES/LTEM system.

In this section, the TES/LTEM system for testing THz emission from graphene/Si samples is introduced. A diagram of the TES/LTEM system is shown in Figure 3. A beam splitter is used to split the femtosecond laser pulses into pump and probe pulses. The pump laser pulses illuminate the sample surface, and THz emission is excited from the illumination area. After that, the generated THz pulse is collimated and focused by an off-axis parabolic mirror and a THz lens onto the photoconductive detector (PCD), which consists of a photoconductive antenna fabricated on a low-temperature-grown gallium arsenide (LT-GaAs) substrate. In our system, a spiral-type antenna is used. To facilitate the detection process, a hemispherical silicon lens was mounted on the front side of the PCD. The THz wave was synchronized with a trigger pulse delivered through a time-delay stage, generating a transient photocurrent at the detector that corresponded to the magnitude of the electric field of the THz pulse. This approach allowed for the efficient detection and analysis of time-resolved THz waveforms. In the experiment, the graphene samples were excited with an 800 nm laser pulse at an incident angle of 30°, and the THz emission was focused on the spiral LT-GaAs THz antenna with variable delays. The beam diameter is as large as 5 mm to observe strong THz emission signals from the samples. The pump power is 200 mW, and the probe power is 5 mW. The sample is mounted onto an x-y stage, which is automatically controlled by a LabView program. The parameters can be easily set from the control window, such as the size of the image and the resolution. In our experiment, the LTEM images were set to be 80 × 80 pixels, and the resolution of the X-Y stages was around 0.3125 mm, which confirms higher mapping quality and can distinguish the large differences between graphene and Si.

## 3. Results and Discussion

**Figure 4:**
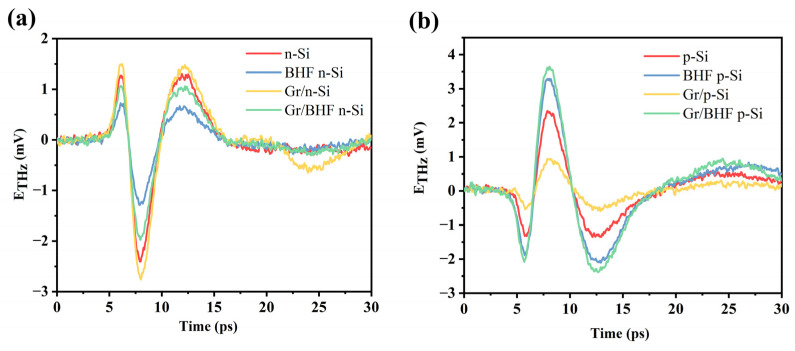
(**a**) THz emission from graphene/n-Si with or without BHF treatment and the comparison with bare n-Si with or without BHF treatment. (**b**) THz emission from graphene/p-Si with or without BHF treatment and the comparison with bare p-Si with or without BHF treatment.

Initially, we assessed the terahertz (THz) emission characteristics from graphene/n-Si and graphene/p-Si samples with and without buffered HF (BHF) treatment, comparing them with THz emissions from bare Si substrates. Figure 4 illustrates that the THz emission amplitude is significantly influenced by the presence of graphene and the surface conditions of the Si substrate. In prior studies, it was noted that the THz emission amplitude of bare n-type Si decreased, while that of p-type Si increased after BHF treatment due to the removal of the native oxide layer and the elevation of the surface-state energy level. Upon transferring graphene onto the n-type Si surface, the THz emission amplitude increased compared to the surface without graphene, regardless of whether the surface underwent BHF treatment or not (Figure 4a). Conversely, when graphene was transferred onto BHF treated p-type Si, the THz emission amplitude decreased compared to that from the bare p-type Si surface without BHF etching (Figure 4b). The variation in THz emission amplitude unveils the impact of graphene on the interface potential of the graphene/Si heterojunction, a factor closely tied to the properties of graphene affected by the surface conditions. When the penetration depth (δ) is larger than the thickness of the interface built-in field, the formula of the THz emission field is expressed as,

(2) $$\;\;\;\;E_{THz}\propto \mu \frac{V_{bi}}{\delta }I_{p},$$

where $E_{THz}$ is the THz emission field, μ is the carrier mobility, $V_{bi}$ is the surface potential, and $I_{p}$ is the intensity of laser power. In the next section, the properties of graphene influenced by the surface conditions will be discussed, and the estimation of graphene via THz emission properties will be introduced.

### 3.1. Influence of Graphene on THz Emission from Graphene/Si Heterojunction

In our previous research, we conducted a systematic examination of THz emissions from Si substrates with varying surface conditions. These differences in surface conditions led to distinct THz emissions in terms of both amplitude and polarity. Importantly, when introducing 2D films like monolayer graphene onto the Si surface, it inevitably influences THz emissions, indicating alterations in the interface’s electrical properties. This influence can be assessed by TES in a non-contact manner. To gain deeper insights into the relationship between THz emission properties and graphene, we conducted measurements on both bare Si surfaces and graphene/Si heterojunctions and compared the results. We also discussed how graphene affects THz emissions in the graphene/Si heterojunction. Extensive research has highlighted the unique Dirac point in the band diagram of monolayer graphene. Depending on the positioning of the Fermi level relative to this point, graphene can be p-doped or n-doped. During the transfer process, CVD graphene comes into contact with photoresist and water for an extended duration, leading to p-doping, and the Fermi level resides below the Dirac point. Moreover, the surface conditions of the substrates inadvertently dope graphene. Consequently, when graphene is transferred onto the Si substrate, the interface properties undergo significant changes, observable from the THz emission spectrum.

The THz emission observed in the graphene/Si heterojunction arises from ultrafast photocarrier transport between graphene and Si, facilitated by surface-band bending. Initially, this band bending occurs due to the pinning effect at the interface, with its magnitude equal to the difference in work function between graphene and Si. The work function represents the energy required for an electron to transition from the Fermi level to the vacuum energy level. For pure Si, this value is approximately 4.85 eV. In doped Si samples, the Fermi level is determined by the type and concentration of doping, causing it to shift away from the mid-gap. This shift alters the initial band bending and subsequently affects the ultrafast photocarrier transport, influencing the THz emission characteristics of the graphene/Si heterojunction. The work function of Si ($\phi_{Si}$) can be calculated as,

(3) $$\phi_{Si}=\chi_{Si}+E_{c}-E_{f},$$

(4) $$\;\;E_{c}-E_{f}=\frac{1}{2}E_{g}\pm kT\ln\frac{N_{D}}{N_{i}},\;$$

where $\chi_{Si}$ is the electron affinity of Si, $E_{f}$ is the Fermi level, $E_{c}$ is the bottom of the conduction band of Si, $E_{g}$ is the band gap of Si, k is the Boltzmann constant, T is the room temperature, $N_{D}$ is the doping concentration, and $N_{i}$ is the intrinsic carrier density. The sign is positive for the p-type and negative for the n-type Si.

**Figure 5:**
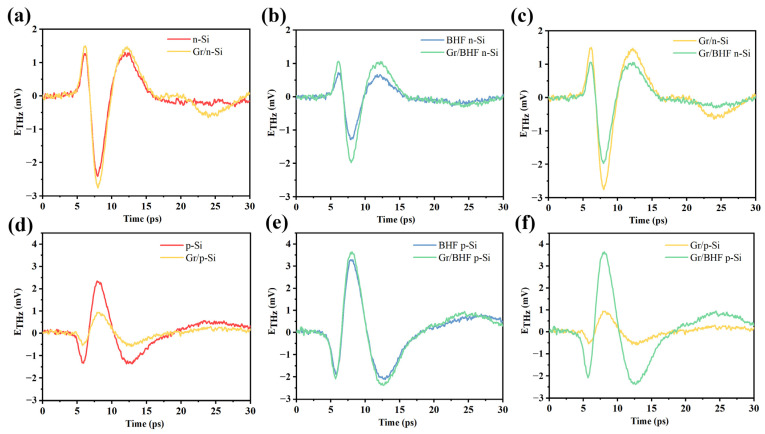
The comparison of the THz emission waveform from different samples (**a**) n-Si vs. Gr/n-Si (**b**) BHF n-Si vs. Gr/BHF n-Si (**c**) Gr/n-Si vs. Gr/BHF n-Si (**d**) p-Si vs. Gr/p-Si (**e**) BHF/p-Si vs. Gr/BHF p-Si (**f**) Gr/p-Si vs. Gr/BHF p-Si.

Figure 5a,d depict the comparison of THz emission with and without graphene on both n-type and p-type Si substrates, respectively. Figure 5b,e further illustrate this comparison of the samples on Si substrates after BHF etching. Based on these experimental findings, we can infer the influence of graphene on THz emissions in the graphene/Si heterojunction. In n-type Si, where the Fermi level is proximate to the conduction band, and in p-type Si, where it is close to the valence band, the work function differs, and the interface-band bending reverses as well. This disparity results in different signs of the THz emission waveforms, as calculated in Table 2. Moreover, upon removal of the native oxide layer, the surface dipoles on bare Si led to a variation in surface-band bending for THz emissions, while in the graphene/Si heterojunction, the work function difference led to the THz emissions. The absolute intensity of the band bending decreases in n-type Si but increases in p-type Si conditions, which mirrors the changes observed in the experimental THz emission intensity.

### 3.2. Influence of Si Surface Conditions on THz Emission from Graphene/Si Heterojunction

The work function of the free-standing graphene is relative to the energy from the Dirac point to the vacuum energy level, which is around 4.5 eV. However, when the graphene is transferred onto the Si surface with a native oxide layer, the graphene will be unintentionally doped to p-type conditions, and the work function of graphene will increase to around 4.8 eV to 5.0 eV. The role of the native oxide layer has been revealed to increase the work function of graphene compared to that on the BHF-treated Si surface. Based on the experimental results, the influence of the removal of the native oxide layer at the Si surface on the THz emission from the graphene/Si heterojunction is concluded. As shown in Figure 5c,f, the waveform is flipping between different doping types of Si substrate. The intensity of THz emissions from graphene/n-type Si is larger than that from graphene/BHF n-type Si, whereas the intensity of THz emissions from graphene/p-type Si is lower than that from graphene/BHF p-type Si. Due to the pinning effect at the interface, band bending occurs, which leads to the acceleration of photocarriers and THz emissions. Owing to the effect of the native oxide layer on the work function of graphene, the band bending is largely changed when the native oxide layer is removed by the BHF. Approximately, the band bending can be calculated by the work function difference between the graphene layer and Si substrate.

**Figure 6:**
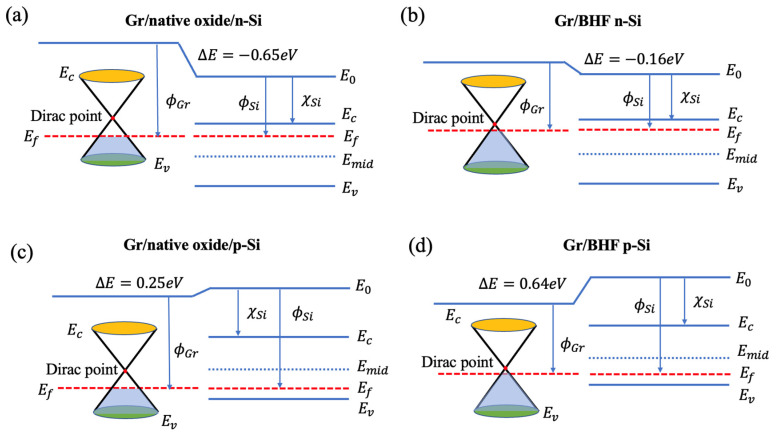
A band diagram of the graphene/Si heterojunction in different situations. (**a**) graphene/native oxide/n-Si, (**b**) graphene/BHF n-Si, (**c**) graphene/native oxide/p-Si, (**d**) graphene/BHF p-Si. The ΔE is equal to the interface potential ($V_{bi}$), where $V_{bi}=\phi_{Si}-\phi_{Gr}$.

**Figure 7:**
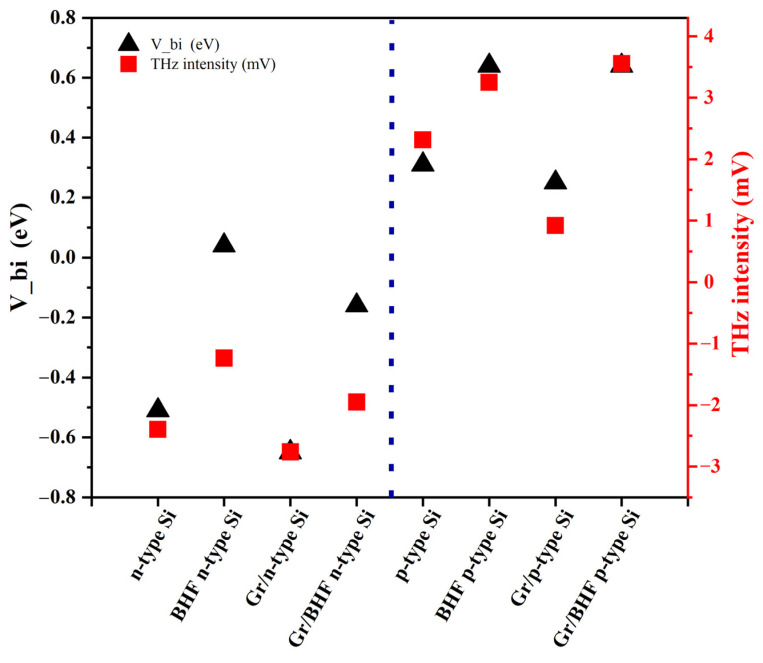
A comparison between the calculated interface-band bending and the THz emission intensity.

Due to the existence of a native oxide layer on the Si surface, hydroxyl groups (–OH) are known to bind to the dangling bonds of Si at the surface and form a hydrophilic layer of silanol (Si-OH) groups. Polar adsorbates such as water molecules easily attach to the Si-OH groups, which results in carrier doping to graphene on the Si substrates [44]. In addition, other charged impurities also easily adsorb at this surface during the experimental processes. These charges also influence the graphene doping conditions and change the work function of graphene. When the graphene is transferred onto the Si surface after removing the native oxide layer by BHF etching, the graphene directly contacts the H-terminated Si surface, and the work function of graphene is slightly increased to around 4.61 eV [45]. With BHF etching on the Si surface, the Si–O bonds are replaced by Si–F bonds and form the F-terminated surface at first as a mid-stage. Subsequently, the Si–Si bonds are inserted by further HF attacks to generate SiF_4_ away from the surface, leaving behind an H-terminated Si surface. The H-terminated Si surface confers stability in the ambient environment. Furthermore, the Si-H groups make the Si surface hydrophobic [46]. Therefore, the doping effect from the water molecules to the graphene is relatively weak compared to the one transferred onto the Si surface with the native oxide layer. Figure 6 shows band diagrams of the graphene/Si heterojunctions in different surface conditions, which successfully explain the flipping of the THz emission waveform and intensity variation due to different doping conditions and BHF etching on the Si surface. As shown in Figure 6a,b, the removal of the native oxide layer on the n-type Si surface reduces the doping effect on graphene, and the work function difference between graphene and Si decreases as well, leading to a decline in the THz emission intensity (Figure 5c). On the other hand, the work function of p-type Si is larger than the graphene, which makes a reversal of the band bending direction as well as a flip in THz emission waveforms compared to the case of n-type Si, as shown in Figure 6c,d. The removal of the native oxide layer on the p-type Si surface increases the difference in work function between graphene and the Si substrate, leading to an increase in THz emission intensity (Figure 5f). Figure 7 illustrates a comparison between the calculated interface-band bending and experimental THz emission intensity of the second peak, and their data are listed in Table 2. We observed significant offsets in the cases of n-type Si treated with BHF. These offsets may be caused by an insufficient BHF etching process conducted over a short period, during which the H-terminated Si surface may not have been fully achieved. Consequently, the surface-state energy level of n-type Si is lower than the calculation theory predicts based on the condition of a fully H-terminated Si surface [30]. Although there are some offsets in BHF n-type Si cases, most of the cases show good agreement between the calculation results and experimental results of THz emission intensity. The experimental and calculated results collectively suggest that the presence of graphene significantly influences THz emission characteristics on Si substrates, and reversely, it can be a potential estimation method for the graphene/Si heterojunction via the THz emission properties.

### 3.3. Mapping the Large-Scale Graphene on Si by LTEM

**Figure 8:**
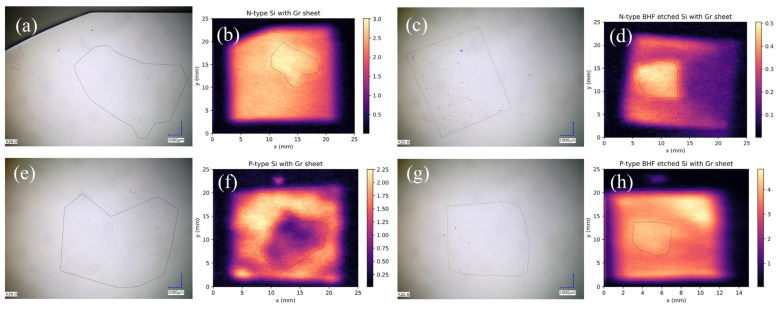
The LTEM images of graphene on the Si substrates compared with optical images. Optical images: (**a**) graphene/n-Si, (**c**) graphene/BHF n-Si, (**e**) graphene/p-Si, (**g**) graphene/BHF p-Si; and the LTEM images: (**b**) graphene/n-Si, (**d**) graphene/BHF n-Si, (**f**) graphene/p-Si, (**h**) graphene/BHF p-Si. The shapes of graphene are shown with the black line in both LTEM images and optical images.

THz emissions present significant potential for assessing monolayer graphene on Si substrates. However, the practice of large-scale graphene mapping necessitates a sensitive x-y stage with an automatic control system. Here, we introduce laser THz emission microscopy (LTEM), a technique that enables the visualization of the distribution of the interface electric field and observation of monolayer graphene on Si substrates. An LTEM image of bare n-type Si is shown in Figure S1, which indicates a homogeneous electric field distribution on the Si surface. Figure 8 illustrates LTEM images of graphene on various types of Si substrates before and after BHF etching, compared with the optical images. By analyzing the influence of graphene on THz emission intensity, we can identify bright areas on n-type Si, with or without BHF etching, and on p-type Si with BHF etching, indicating the presence of the graphene layer. Conversely, dark areas on p-type Si without BHF etching correspond to the graphene layer. In this comparison, we illustrated the disparities between optical and LTEM images. The optical images struggle to depict graphene due to its atomic thickness and high transparency. Conversely, LTEM imaging proves highly effective in discerning the graphene distribution on the Si surface, primarily because of graphene’s substantial impact on the interface electric field.

The experimental results demonstrate a noticeable difference in THz emission between areas with and without graphene, affirming the feasibility of large-scale mapping using LTEM. However, there are still significant challenges ahead. Firstly, improvements are needed to enhance the resolution for detailed mapping of smaller graphene structures. The spatial resolution of LTEM is contingent upon the beam spot size of the incident laser pump. However, reducing the beam spot size often leads to a decrease in THz emission intensity, posing challenges in detection. Overcoming this hurdle requires enhancing the measurement environment and detector sensitivity while minimizing noise. Addressing these issues will be crucial in advancing the capabilities of LTEM for high-resolution mapping of graphene structures, thus enabling more comprehensive characterization and utilization of graphene in electronic devices. Secondly, the wafer-scale mapping requires an ultrafast large-scale scanning X-Y stage to reduce the mapping time for rapid evaluation. Furthermore, THz emissions from the graphene/Si heterojunction are significantly influenced by the conditions of the Si surface, which may, in turn, affect the properties of graphene itself. Therefore, further research is required to enhance the spatial resolution and achieve accurate estimation of graphene/Si heterojunctions using the LTEM system. In the future, LTEM is poised to become a valuable method for imaging CVD graphene on Si substrates with higher resolution and faster estimation speeds, thereby supporting the study and development of graphene-based devices.

## 4. Conclusions

In conclusion, we utilized terahertz emission spectroscopy and microscopy (TES/LTEM) to assess large-scale chemical vapor deposition (CVD) monolayer graphene transferred onto silicon substrates. We evaluated the dynamic electronic properties of graphene and performed large-scale graphene mapping. By comparing the THz emission properties from monolayer graphene on different types of silicon substrates, including those treated with buffered oxide etches, we gained insights into the influence of native oxide layers and surface dipoles on graphene behavior. Furthermore, we discussed the mechanism of THz emissions from the graphene/silicon heterojunction, shedding light on the intricate interplay between these materials. Ultimately, we successfully achieved large-scale mapping of monolayer graphene on silicon, showcasing the efficacy of TES/LTEM for graphene characterization in the modern graphene-based semiconductor industry. These findings underscore the potential of terahertz-based techniques in advancing graphene integration and fabrication processes, paving the way for innovative electronic devices with enhanced functionalities.

## Figures and Tables

**Table 1:** The properties of the Si substrates.

	p-Si	n-Si
Dopant density (cm^−3^)	1 × 10^17^	5 × 10^16^
Resistivity range (Ω·cm)	0.1~1	0.1~1

**Table 2:** Calculated band bending ($V_{bi}$) vs. experiment THz emission intensity.

	*V_bi_* (eV)	THz Intensity (mV)
n-type Si	−0.51	−2.398
p-type Si	0.31	2.312
BHF n-type Si	0.04	−1.238
BHF p-type Si	0.64	3.247
Gr/n-type Si	−0.65	−2.762
Gr/p-type Si	0.25	0.919
Gr/BHF n-type Si	−0.16	−1.949
Gr/BHF p-type Si	0.64	3.552

## Data Availability

The data that support the findings of this study are available from the corresponding author upon reasonable request.

## Supplementary Materials

The following supporting information can be downloaded at: https://www.mdpi.com/article/10.3390/ma17071497/s1, Figure S1: The LTEM image of n-Si substrate without graphene transfer.
