# Supplementary material for: Sensitive Characterization of the Graphene Transferred onto Varied Si Wafer Surfaces via Terahertz Emission Spectroscopy and Microscopy (TES/LTEM)
Source: Materials (Basel). 2024 Mar 26;17(7):1497. doi: 10.3390/ma17071497 (PMC11012325; doi:10.3390/ma17071497)
Supplement: Supplementary file 1 [file materials-17-01497-s001.zip › materials-2913334-supplementary.pdf]

The LTEM images of Si substrate before transferring graphene.

Here, we provide the LTEM image of n-Si before graphene transferring and BHF etching. The result shows the homogeneous electric field distribution on the Si surface.

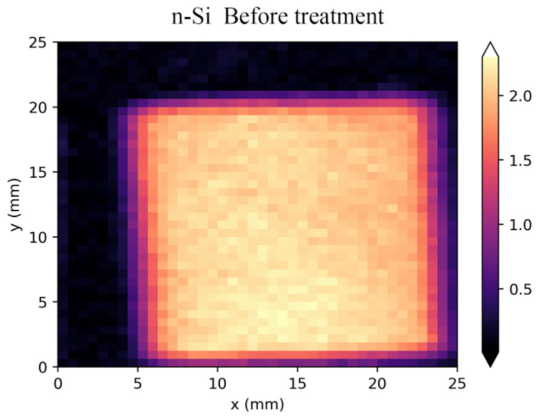

Figure S1. The LTEM image of n-Si substrate without graphene transfer.
